# Comparison of the crystal structures of methyl 4-bromo-2-(meth­oxy­meth­oxy)benzoate and 4-bromo-3-(meth­oxy­meth­oxy)benzoic acid

**DOI:** 10.1107/S2056989016003777

**Published:** 2016-03-11

**Authors:** P. A. Suchetan, V. Suneetha, S. Naveen, N. K. Lokanath, P. Krishna Murthy

**Affiliations:** a Department of Chemistry, University College of Science, Tumkur University, Tumkur 572 103, India; b Department of Chemistry, Bapatla Engineering College (Autonomous), Bapatla A. P., India; c Institution of Excellence, University of Mysore, Manasagangotri, Mysuru-6, India; d Department of Physics, University of Mysore, Manasagangotri, Mysuru-6, India

**Keywords:** crystal structure, bromo hy­droxy benzoic acids, C—H⋯π_ar­yl_ inter­actions, π–π inter­actions, hydrogen bonding

## Abstract

The title compounds, C_10_H_11_BrO_4_, (I), and C_9_H_9_BrO_4_, (II), are derivatives of bromo–hy­droxy–benzoic acids. Compound (II) crystallizes with two independent mol­ecules (*A* and *B*) in the asymmetric unit. In both (I) and (II), the O—CH_2_—O—CH_3_ side chain is not in its fully extended conformation; the O—C—O—C torsion angle is 67.3 (3) ° in (I), and −65.8 (3) and −74.1 (3)° in mol­ecules *A* and *B*, respectively, in compound (II). In the crystal of (I), mol­ecules are linked by C—H⋯O hydrogen bonds, forming *C*(5) chains along [010]. The chains are linked by short Br⋯O contacts [3.047 (2) Å], forming sheets parallel to the *bc* plane. The sheets are linked *via* C—H⋯π inter­actions, forming a three-dimensional architecture. In the crystal of (II), mol­ecules *A* and *B* are linked to form *R*_2_^2^(8) dimers *via* two strong O—H⋯O hydrogen bonds. These dimers are linked into ⋯*A*–*B*⋯*A*–*B*⋯*A*–*B*⋯ [*C*_2_^2^(15)] chains along [011] by C—H⋯O hydrogen bonds. The chains are linked by slipped parallel π–π inter­actions [inter-centroid distances = 3.6787 (18) and 3.8431 (17) Å], leading to the formation of slabs parallel to the *bc* plane.

## Abstract 2

The crystal structures of two bromo–hy­droxy–benzoic acid derivatives, namely, methyl 4-bromo-2-(meth­oxy­meth­oxy)benzoate, (I), and 4-bromo-3-(meth­oxy­meth­oxy)benzoic acid, (II), are compared. Compound (II) crystallizes with two independent mol­ecules in the asymmetric unit. In the crystal structures of both compounds, two-dimensional architectures are formed principally by C—H⋯O hydrogen bonds, and by Br⋯O inter­actions in (I) and by π–π inter­actions in (II).

## Chemical context   

Ester derivatives of many compounds exhibit a variety of pharmacological properties, such as anti­cancer, anti­tumor and anti­microbial activities (Anadu *et al.*, 2006 ▸; Bartzatt *et al.*, 2004 ▸; Bi *et al.*, 2012 ▸). Salicylic acid and derivatives of salicylic acid are of great biological importance. For example, they are known for their analgesic and anti-inflammatory activities in the treatment of rheumatoid arthritis (Anderson *et al.*, 2014 ▸; Hardie, 2013 ▸). They are also known for their use as anti­bacterial and anti­mycobacterial agents (Silva *et al.*, 2008 ▸). In view of the above, compounds (I) and (II) were synthesized and we report herein on their crystal structures.
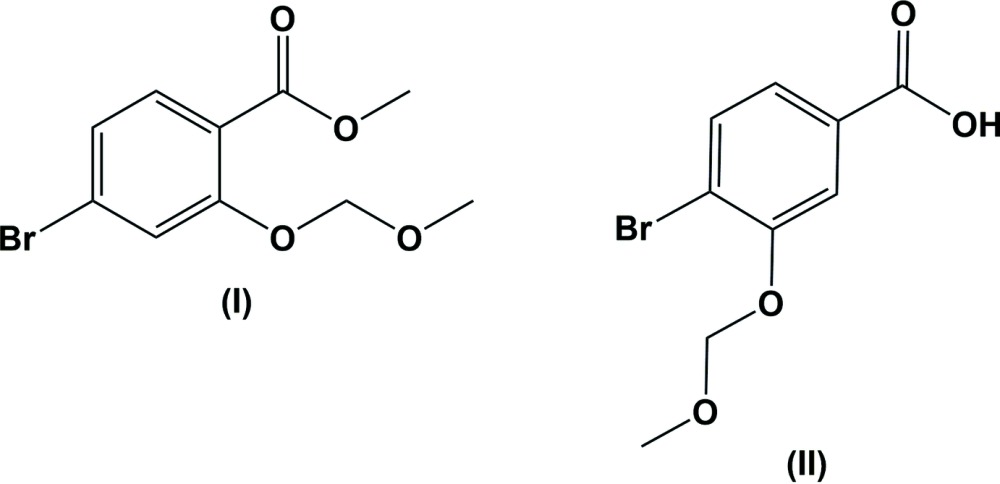


## Structural commentary   

**Figure 1:**
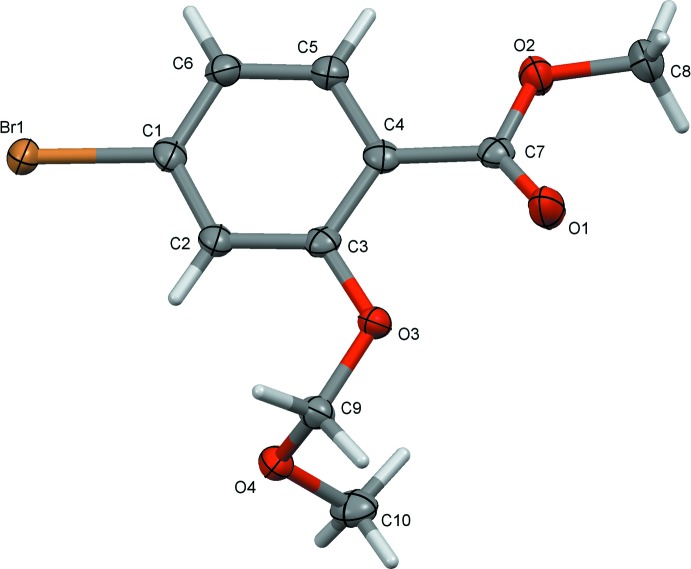
A view of the mol­ecular structure of compound (I), showing the atom labelling. Displacement ellipsoids are drawn at the 50% probability level.

The mol­ecular structure of compound (I), is illustrated in Fig. 1 ▸. The –O–CH_2_–O-CH_3_ side chain is not in its fully extended conformation, with torsion angle O3—C9—O4—C10 being 67.3 (3)°. The dihedral angle between the benzene ring and the ester segment (O1/C7/O2/C8) is 14.5 (2)°, while the plane through atoms C10/O4/C9 of the meth­oxy­meth­oxy side chain is inclined to the benzene ring by 82.5 (3)°.

**Figure 2:**
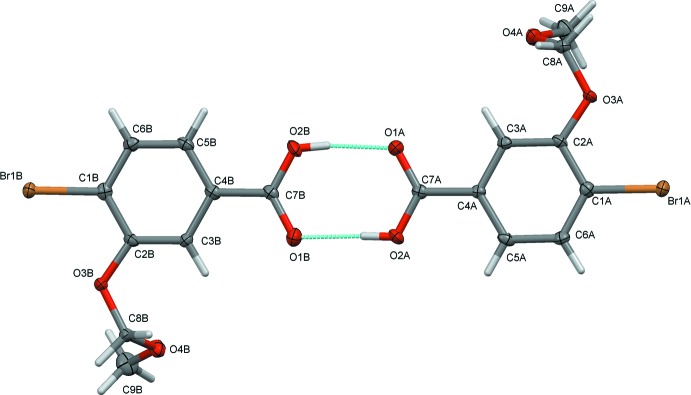
A view of the mol­ecular structure of compound (II), showing the atom labelling. Displacement ellipsoids are drawn at the 50% probability level. O—H⋯O hydrogen bonds are shown as dashed lines (see Table 2 ▸).

**Figure 3:**
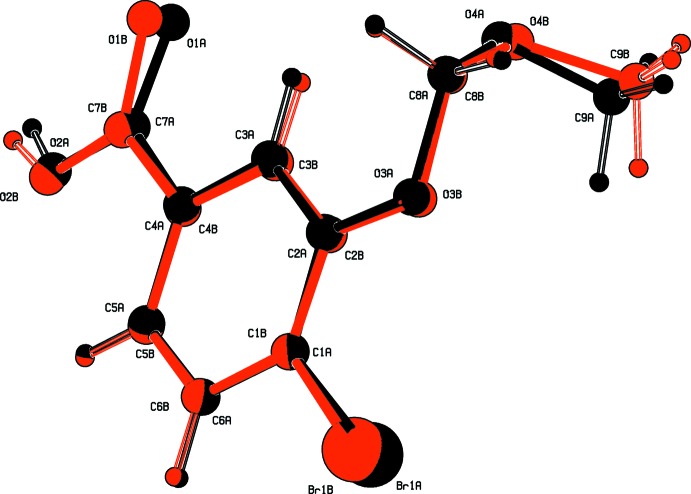
A view of the mol­ecular fit of mol­ecules *A* (black) and *B* (red) of compound (II).

The mol­ecular structure of compound (II), is illustrated in Fig. 2 ▸. It crystallizes with two independent mol­ecules (*A* and *B*) in the asymmetric unit. The conformations of the two mol­ecules differ in the torsion angles of the –O–CH_2_–O–CH_3_ side chains and the orientation of the –COO– group with respect to the benzene ring, as shown in the *AutoMolFit* diagram (Fig. 3 ▸; Spek, 2009 ▸). The –O–CH_2_–O–CH_3_ side chains in mol­ecules *A* and *B* are not in their fully extended conformation; torsion angle O3*A*—C8*A*—O4*A*—C9*A* in mol­ecule *A* is −65.8 (3)°, and torsion angle O3*B*—C8*B*—O4*B–*-C9*B* in mol­ecule *B* is −74.1 (3)°. The dihedral angle between the benzene ring and the plane through atoms C8*A*/O4*A*/C9*A* of the meth­oxy­meth­oxy side chain in mol­ecule *A* is 79.2 (3)°, while the corresponding dihedral angle in mol­ecule *B*, between the benzene ring and plane C9*B*/O4*B*/O8*B* is 67.1 (3)°. This is less than in compound (I) and further, the dihedral angle between the benzene ring and the –COO– group is 6.6 (4)° in *A* and 9.1 (4)° in *B*; also less than observed in compound (I), *viz.* 14.5 (2)°.

## Supra­molecular features   

**Figure 4:**
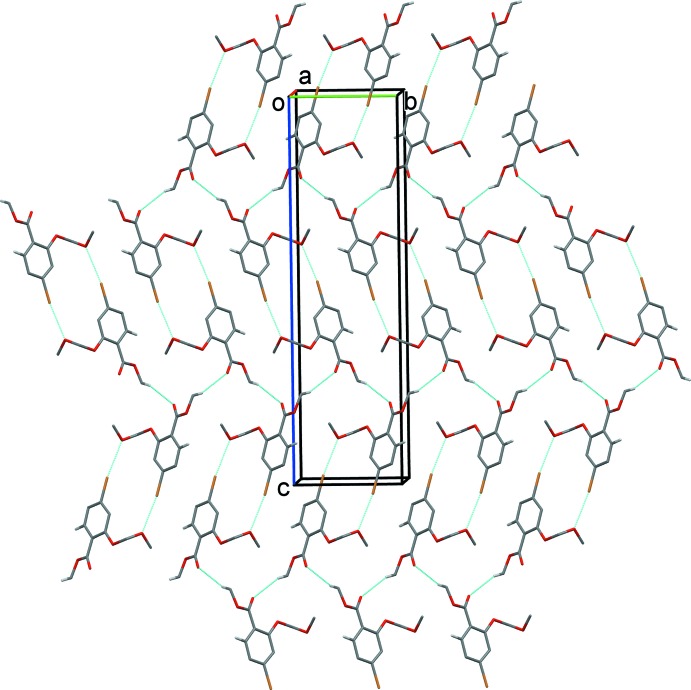
A view along the *a* axis of the crystal packing of compound (I). C—H⋯O and Br⋯O inter­actions are shown as dashed lines (see Table 1 ▸). H atoms not involved in these inter­actions have been omitted for clarity.

**Figure 5:**
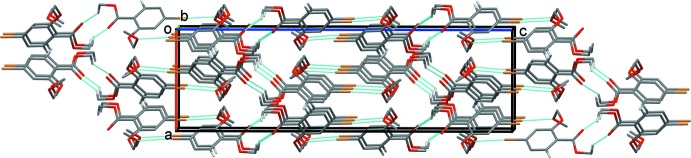
A view along the *b* axis of the crystal packing of compound (I). C—H⋯O and Br⋯O inter­actions are shown as dashed lines (see Table 1 ▸). H atoms not involved in these inter­actions have been omitted for clarity.

In the crystal of (I), mol­ecules are linked by structure-directing C8—H8*A*⋯O1 hydrogen bonds (Table 1 ▸ and Fig. 4 ▸), forming *C*(5) chains along the *b* axis. Adjacent chains are linked by short Br1⋯O4^i^ contacts [*d*_Br⋯O_ = 3.047 (2) Å; symmetry code (i): −*x*, −*y*, −*z* + 1] leading to the formation of sheets parallel to plane (100). The sheets are linked by C5—H5⋯π inter­actions (centroid of the benzene ring C1–C6) along the *a-*axis direction, forming a three-dimensional structure (Table 1 ▸ and Fig. 5 ▸).

**Figure 6:**
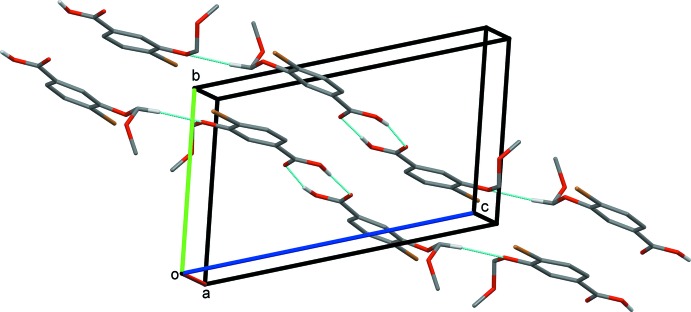
A partial view along the *a* axis of the crystal packing of compound (II). O—H⋯O and C—-H⋯O hydrogen bonds are shown as dashed lines (see Table 2 ▸). H atoms not involved in these inter­actions have been omitted for clarity.

**Figure 7:**
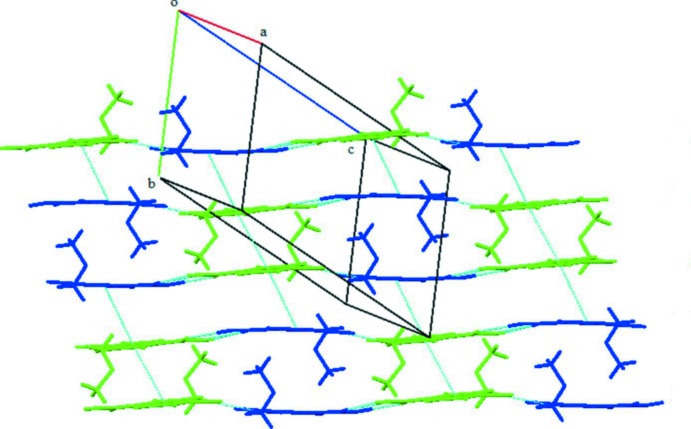
A view of the π–π stacking observed in the crystal of (II); mol­ecule *A* green, mol­ecule *B* blue.

In the crystal of (II), mol­ecules *A* and *B* are linked *via* two strong O—H⋯O hydrogen bonds, namely, O2*A–*-H2*A*⋯O1*B* and O2*B–*-H2*B*⋯O1*A*, forming dimers with an 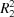
(8) ring motif (Table 2 ▸ and Fig. 6 ▸). Adjacent dimers are linked by C8*B*—H8*B*2⋯·O3*A* hydrogen bonds (Table 2 ▸), forming chains along [011]. The chains are linked *via* slipped parallel π–π inter­actions between *B* mol­ecules [*Cg*2⋯*Cg*2^ii^ distance = 3.6792 (18) Å; *Cg*2 is the centroid of ring C1*B*–C6*B*; inter-planar distance = 3.3691 (12) Å; slippage = 1.477 Å; symmetry code (ii): −*x*, −*y* + 2, −*z* + 1], and between *A* and *B* mol­ecules [*Cg*1⋯*Cg*2^iii^ = 3.8431 (17) Å; *Cg*1 is the centroid of the ring C1*A*–C6*A*; inter-planar distance = 3.5538 (12) Å; slippage 1.98 Å; symmetry code (iii): −*x* + 1, −*y* + 1, −*z* + 1], thus forming slabs lying parallel to the *bc* plane (Fig. 7 ▸).

## Synthesis and crystallization   

**Synthesis of methyl 4-bromo-2-(meth­oxy­meth­oxy) benzoate (I)**

To a stirred solution of methyl 4-bromo-2-hy­droxy-benzoate (1.0 g, 4.32 mmol) in di­chloro­methane (15 ml) (DCM) was added *N*,*N*-diiso­propyl­ethyl­amine (1.5 ml, 8.65 mmol) (DIPEA), followed by chloro­methyl methyl ether (0.49 ml, 6.49 mmol) (MOM-Cl), at 273 K and the reaction mixture was stirred at room temperature overnight. The reaction mixture was then diluted with water (50 ml) and the organic layer was extracted with ethyl acetate (2 × 50 ml). The combined organic layers were washed successively with water, brine, dried over anhydrous magnesium sulfate (MgSO_4_), filtered and the filtrate was concentrated under reduced pressure. The crude product was purified by column chromatography using ethyl acetate:hexane (1:9) as eluent to afford (I) as an off-white coloured solid (yield: 0.851g, 71.4%; m.p.: 353 K). ^1^H NMR (DMSO-*d*_6_, 400 MHz, p.p.m.): δ = 3.39 (3H, *s*), 3.79 (3H, *s*), 5.29 (2H, *s*) 7.29 (1H, *dd*, *J* = 1.20 Hz, 1.20 Hz), 7.44 (1H, *s*), 7.60 (1H, *d*, *J* = 8.00 Hz).

**Synthesis of 4-bromo-3-(meth­oxy­meth­oxy)benzoic acid (II)**

A mixture of methyl 4-bromo-3-(meth­oxy­meth­oxy) benzoate (1 g, 3.63 mmol), 10% aqueous potassium hydroxide (0.61 g, 3.0 mmol), tetra­hydro­furan (5 ml) and methanol (20 ml) was stirred at room temperature for 2 h. The mixture was then concentrated to remove organic solvents and the aqueous layer was acidified with 6 *N* hydro­chloric acid. The precipitated solid was filtered, dried under vacuum to afford (II) as a white solid (yield: 0.86g, 91%; m.p.: 433 K). ^1^H NMR (DMSO-*d*_6_, 400 MHz, p.p.m.): δ = 3.39 (3H, *s*), 5.28 (2H, *s*), 7.26 (1H, *dd*, *J* = 1.20 Hz, 1.20 Hz), 7.40 (1H, *s*), 7.59 (1H, *d*, *J* = 8.00 Hz), 12.90 (1H, *s*).

Single crystals of compounds (I) and (II), suitable for X-ray diffraction studies, were obtained by solvent evaporation using methanol:chloro­form (2:1) as the solvent mixture.

## Refinement details   

Crystal data, data collection and structure refinement details are summarized in Table 3 ▸. The H atoms of the OH groups in (II) were located in a difference Fourier map and refined with a distance restraint: O—H = 0.84 (5) Å. The C-bound H atoms in (I) and (II) were positioned with idealized geometry and refined using a riding model: C—H = 0.95–0.99 Å, with *U*_iso_(H) = 1.5*U*_eq_(C-meth­yl) and 1.2*U*_eq_(C) for other H atoms. In the final cycles of refinement reflection (0 0 2) in (I) and reflections (4 1 0), (6 − 4 6), (5 − 5 7), (4 2 0) and (0 − 1 6) in (II) were omitted due to large differences in *F* ^2^_obs_ and *F* ^2^_calc_, considerably improving the values of *R*1, *wR*2, and GOF.

## Supplementary Material

Crystal structure: contains datablock(s) I, II, Global. DOI: 10.1107/S2056989016003777/su5284sup1.cif

Supporting information file. DOI: 10.1107/S2056989016003777/su5284Isup2.cml

Supporting information file. DOI: 10.1107/S2056989016003777/su5284IIsup3.cml

CCDC references: 1457944, 1457943

Additional supporting information:  crystallographic information; 3D view; checkCIF report

## Figures and Tables

**Table 1:** Hydrogen-bond geometry (Å, °) for (I) *Cg*1 is the centroid of the C1–C6 benzene ring.

*D*—H⋯*A*	*D*—H	H⋯*A*	*D*⋯*A*	*D*—H⋯*A*
C8—H8*A*⋯O1^i^	0.98	2.58	3.439 (5)	147
C5—H5⋯*Cg*1^ii^	0.95	2.95	3.765 (4)	129

Symmetry codes: (i) 
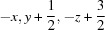
; (ii) 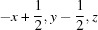.

**Table 2:** Hydrogen-bond geometry (Å, °) for (II)

*D*—H⋯*A*	*D*—H	H⋯*A*	*D*⋯*A*	*D*—H⋯*A*
O2*A*—H2*A*⋯O1*B*	0.84 (5)	1.80 (5)	2.635 (4)	178 (5)
O2*B*—H2*B*⋯O1*A*	0.82 (5)	1.81 (5)	2.621 (4)	167 (5)
C8*B*—H8*B*2⋯O3*A* ^i^	0.99	2.52	3.420 (4)	150

Symmetry code: (i) 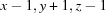.

**Table 3:** Experimental details

	(I)	(II)
Crystal data		
Chemical formula	C_10_H_11_BrO_4_	C_9_H_9_BrO_4_
*M*_r_	275.10	261.07
Crystal system, space group	Orthorhombic, *P* *b* *c* *a*	Triclinic, *P* 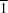
Temperature (K)	173	173
*a*, *b*, *c* (Å)	8.8487 (13), 8.1514 (11), 29.284 (4)	7.7211 (3), 9.6881 (4), 14.2627 (6)
α, β, γ (°)	90, 90, 90	73.635 (1), 77.664 (1), 69.577 (1)
*V* (Å^3^)	2112.2 (5)	951.40 (7)
*Z*	8	4
Radiation type	Cu *K*α	Cu *K*α
μ (mm^−1^)	5.27	5.82
Crystal size (mm)	0.29 × 0.22 × 0.19	0.28 × 0.25 × 0.22

Data collection		
Diffractometer	Bruker APEXII	Bruker APEXII
Absorption correction	Multi-scan (*SADABS*; Bruker, 2009 ▸)	Multi-scan (*SADABS*; Bruker, 2009 ▸)
*T*_min_, *T*_max_	0.286, 0.367	0.245, 0.278
No. of measured, independent and observed [*I* > 2σ(*I*)] reflections	8999, 1751, 1720	11112, 3031, 2930
*R*_int_	0.052	0.040
(sin θ/λ)_max_ (Å^−1^)	0.590	0.585

Refinement		
*R*[*F* ^2^ > 2σ(*F* ^2^)], *wR*(*F* ^2^), *S*	0.052, 0.139, 1.14	0.039, 0.120, 1.09
No. of reflections	1751	3031
No. of parameters	138	261
No. of restraints	0	2
H-atom treatment	H-atom parameters constrained	H atoms treated by a mixture of independent and constrained refinement
Δρ_max_, Δρ_min_ (e Å^−3^)	1.87, −0.97	0.67, −1.08

Computer programs: *APEX2*, *SAINT-Plus* and *XPREP* (Bruker, 2009 ▸), *SHELXS97* and *SHELXL97* (Sheldrick, 2008 ▸) and *Mercury* (Macrae *et al.*, 2008 ▸).

## supplementary crystallographic information

### Crystal data

**Table:**

C_9_H_9_BrO_4_	*Z* = 4
*M**_r_* = 261.07	*F*(000) = 520
Triclinic, *P*1	*D*_x_ = 1.823 Mg m^−^^3^
Hall symbol: -P 1	Melting point: 433 K
*a* = 7.7211 (3) Å	Cu *K*α radiation, λ = 1.54178 Å
*b* = 9.6881 (4) Å	Cell parameters from 123 reflections
*c* = 14.2627 (6) Å	θ = 3.3–64.4°
α = 73.635 (1)°	µ = 5.82 mm^−^^1^
β = 77.664 (1)°	*T* = 173 K
γ = 69.577 (1)°	Prism, colourless
*V* = 951.40 (7) Å^3^	0.28 × 0.25 × 0.22 mm

### Data collection

**Table:**

Bruker APEXII diffractometer	3031 independent reflections
Radiation source: fine-focus sealed tube	2930 reflections with *I* > 2σ(*I*)
Graphite monochromator	*R*_int_ = 0.040
phi and φ scans	θ_max_ = 64.4°, θ_min_ = 3.3°
Absorption correction: multi-scan (*SADABS*; Bruker, 2009)	*h* = −8→8
*T*_min_ = 0.245, *T*_max_ = 0.278	*k* = −11→11
11112 measured reflections	*l* = −15→16

### Refinement

**Table:**

Refinement on *F*^2^	Primary atom site location: structure-invariant direct methods
Least-squares matrix: full	Secondary atom site location: difference Fourier map
*R*[*F*^2^ > 2σ(*F*^2^)] = 0.039	Hydrogen site location: inferred from neighbouring sites
*wR*(*F*^2^) = 0.120	H atoms treated by a mixture of independent and constrained refinement
*S* = 1.09	*w* = 1/[σ^2^(*F*_o_^2^) + (0.0844*P*)^2^ + 0.9411*P*] where *P* = (*F*_o_^2^ + 2*F*_c_^2^)/3
3031 reflections	(Δ/σ)_max_ = 0.001
261 parameters	Δρ_max_ = 0.67 e Å^−^^3^
2 restraints	Δρ_min_ = −1.08 e Å^−^^3^

### Special details

**Table:**

Geometry. All s.u.'s (except the s.u. in the dihedral angle between two l.s. planes) are estimated using the full covariance matrix. The cell s.u.'s are taken into account individually in the estimation of s.u.'s in distances, angles and torsion angles; correlations between s.u.'s in cell parameters are only used when they are defined by crystal symmetry. An approximate (isotropic) treatment of cell s.u.'s is used for estimating s.u.'s involving l.s. planes.
Refinement. Refinement of *F*^2^ against ALL reflections. The weighted *R*-factor *wR* and goodness of fit *S* are based on *F*^2^, conventional *R*-factors *R* are based on *F*, with *F* set to zero for negative *F*^2^. The threshold expression of *F*^2^ > 2σ(*F*^2^) is used only for calculating *R*-factors(gt) *etc*. and is not relevant to the choice of reflections for refinement. *R*-factors based on *F*^2^ are statistically about twice as large as those based on *F*, and *R*- factors based on ALL data will be even larger.

### Fractional atomic coordinates and isotropic or equivalent isotropic displacement parameters (Å^2^)

**Table:**

	*x*	*y*	*z*	*U*_iso_*/*U*_eq_	
Br1B	−0.40349 (5)	1.06722 (4)	0.30367 (2)	0.01682 (17)	
Br1A	1.35506 (5)	0.17904 (4)	0.90000 (2)	0.01972 (17)	
O3B	−0.0105 (3)	1.0670 (3)	0.23290 (16)	0.0159 (5)	
O3A	0.9612 (3)	0.1908 (2)	0.96819 (15)	0.0164 (5)	
O4B	0.2558 (3)	1.1441 (2)	0.20751 (16)	0.0204 (5)	
O4A	0.6543 (3)	0.3227 (3)	1.02268 (16)	0.0223 (5)	
O1A	0.5360 (3)	0.4837 (3)	0.70491 (16)	0.0210 (5)	
O2A	0.7281 (3)	0.5390 (3)	0.56876 (16)	0.0184 (5)	
O1B	0.4376 (3)	0.7142 (3)	0.47778 (18)	0.0219 (5)	
O2B	0.2466 (4)	0.6594 (3)	0.61509 (17)	0.0220 (5)	
C4A	0.8598 (4)	0.3942 (3)	0.7147 (2)	0.0113 (6)	
C7B	0.2753 (5)	0.7246 (3)	0.5251 (2)	0.0141 (6)	
C4B	0.1108 (5)	0.8140 (3)	0.4734 (2)	0.0138 (7)	
C2A	0.9761 (5)	0.2599 (3)	0.8698 (2)	0.0135 (6)	
C7A	0.6971 (5)	0.4759 (3)	0.6597 (2)	0.0125 (6)	
C5A	1.0392 (5)	0.3920 (3)	0.6689 (2)	0.0150 (7)	
H5A	1.0601	0.4358	0.6006	0.018*	
C3A	0.8282 (5)	0.3270 (3)	0.8147 (2)	0.0134 (6)	
H3A	0.7058	0.3274	0.8447	0.016*	
C3B	0.1349 (5)	0.9014 (3)	0.3777 (2)	0.0127 (6)	
H3B	0.2555	0.9065	0.3476	0.015*	
C2B	−0.0179 (5)	0.9803 (3)	0.3272 (2)	0.0133 (7)	
C5B	−0.0660 (5)	0.8083 (3)	0.5191 (2)	0.0153 (7)	
H5B	−0.0822	0.7511	0.5846	0.018*	
C6B	−0.2167 (5)	0.8863 (3)	0.4684 (2)	0.0153 (7)	
H6B	−0.3375	0.8823	0.4987	0.018*	
C1B	−0.1937 (5)	0.9706 (3)	0.3735 (2)	0.0148 (7)	
C6A	1.1867 (5)	0.3251 (3)	0.7243 (2)	0.0155 (7)	
H6A	1.3099	0.3221	0.6942	0.019*	
C9A	0.7006 (6)	0.3967 (4)	1.0828 (3)	0.0285 (8)	
H9A1	0.7188	0.3296	1.1481	0.043*	
H9A2	0.5993	0.4899	1.0894	0.043*	
H9A3	0.8157	0.4213	1.0522	0.043*	
C8A	0.7818 (5)	0.1825 (4)	1.0171 (2)	0.0174 (7)	
H8A1	0.7332	0.1324	0.9816	0.021*	
H8A2	0.7952	0.1192	1.0847	0.021*	
C8B	0.1691 (5)	1.0616 (4)	0.1787 (2)	0.0154 (7)	
H8B1	0.2491	0.9549	0.1876	0.018*	
H8B2	0.1561	1.1016	0.1077	0.018*	
C1A	1.1533 (5)	0.2628 (3)	0.8237 (2)	0.0155 (7)	
C9B	0.1791 (6)	1.3037 (4)	0.1746 (3)	0.0292 (8)	
H9B1	0.1718	1.3290	0.1037	0.044*	
H9B2	0.2588	1.3541	0.1875	0.044*	
H9B3	0.0539	1.3378	0.2100	0.044*	
H2A	0.634 (6)	0.593 (5)	0.541 (3)	0.035*	
H2B	0.330 (6)	0.612 (5)	0.650 (3)	0.035*	

### Atomic displacement parameters (Å^2^)

**Table:**

	*U*^11^	*U*^22^	*U*^33^	*U*^12^	*U*^13^	*U*^23^
Br1B	0.0115 (3)	0.0212 (2)	0.0153 (2)	−0.00265 (17)	−0.00350 (16)	−0.00218 (16)
Br1A	0.0121 (3)	0.0260 (2)	0.0181 (2)	−0.00483 (17)	−0.00408 (16)	−0.00011 (16)
O3B	0.0121 (12)	0.0222 (11)	0.0109 (11)	−0.0052 (10)	−0.0023 (9)	0.0005 (9)
O3A	0.0138 (12)	0.0219 (12)	0.0085 (10)	−0.0029 (10)	−0.0007 (9)	0.0005 (9)
O4B	0.0222 (13)	0.0189 (11)	0.0203 (12)	−0.0083 (10)	−0.0063 (9)	0.0003 (9)
O4A	0.0164 (13)	0.0267 (12)	0.0164 (11)	0.0021 (10)	−0.0016 (9)	−0.0052 (9)
O1A	0.0144 (14)	0.0289 (12)	0.0159 (11)	−0.0062 (11)	−0.0022 (9)	0.0004 (9)
O2A	0.0200 (13)	0.0212 (11)	0.0105 (11)	−0.0058 (10)	−0.0034 (9)	0.0023 (9)
O1B	0.0158 (13)	0.0243 (12)	0.0233 (12)	−0.0044 (10)	−0.0052 (10)	−0.0018 (10)
O2B	0.0237 (14)	0.0244 (12)	0.0149 (12)	−0.0091 (11)	−0.0088 (10)	0.0067 (9)
C4A	0.0135 (17)	0.0104 (13)	0.0110 (14)	−0.0045 (12)	−0.0002 (12)	−0.0042 (11)
C7B	0.0193 (18)	0.0122 (14)	0.0120 (15)	−0.0055 (13)	−0.0022 (12)	−0.0036 (11)
C4B	0.0182 (18)	0.0120 (14)	0.0125 (15)	−0.0053 (13)	−0.0016 (12)	−0.0042 (11)
C2A	0.0177 (18)	0.0124 (14)	0.0092 (15)	−0.0037 (13)	−0.0008 (12)	−0.0022 (12)
C7A	0.0156 (17)	0.0110 (14)	0.0125 (15)	−0.0046 (12)	−0.0007 (12)	−0.0053 (11)
C5A	0.0194 (18)	0.0115 (14)	0.0113 (14)	−0.0028 (13)	0.0009 (12)	−0.0027 (11)
C3A	0.0137 (17)	0.0145 (14)	0.0130 (15)	−0.0063 (13)	−0.0002 (12)	−0.0032 (12)
C3B	0.0108 (16)	0.0137 (14)	0.0137 (15)	−0.0039 (12)	−0.0006 (12)	−0.0038 (12)
C2B	0.0173 (18)	0.0143 (14)	0.0096 (15)	−0.0066 (13)	−0.0016 (12)	−0.0025 (11)
C5B	0.0214 (18)	0.0152 (14)	0.0102 (14)	−0.0073 (13)	−0.0012 (12)	−0.0025 (11)
C6B	0.0153 (18)	0.0180 (15)	0.0151 (15)	−0.0067 (13)	0.0005 (12)	−0.0073 (12)
C1B	0.0169 (18)	0.0116 (14)	0.0166 (16)	−0.0022 (13)	−0.0034 (13)	−0.0061 (12)
C6A	0.0147 (18)	0.0166 (15)	0.0161 (15)	−0.0076 (13)	0.0015 (13)	−0.0044 (12)
C9A	0.033 (2)	0.0260 (18)	0.0227 (18)	−0.0002 (16)	−0.0086 (15)	−0.0083 (14)
C8A	0.0133 (18)	0.0233 (16)	0.0134 (15)	−0.0059 (14)	−0.0005 (12)	−0.0013 (12)
C8B	0.0127 (17)	0.0217 (16)	0.0125 (15)	−0.0066 (13)	−0.0011 (12)	−0.0038 (12)
C1A	0.0160 (18)	0.0133 (14)	0.0158 (16)	−0.0020 (13)	−0.0020 (13)	−0.0041 (12)
C9B	0.037 (2)	0.0185 (16)	0.033 (2)	−0.0084 (16)	−0.0077 (16)	−0.0052 (14)

### Geometric parameters (Å, º)

**Table:**

Br1B—C1B	1.897 (3)	C2A—C1A	1.391 (5)
Br1A—C1A	1.901 (3)	C5A—C6A	1.387 (5)
O3B—C2B	1.371 (4)	C5A—H5A	0.9500
O3B—C8B	1.428 (4)	C3A—H3A	0.9500
O3A—C2A	1.374 (4)	C3B—C2B	1.385 (5)
O3A—C8A	1.430 (4)	C3B—H3B	0.9500
O4B—C8B	1.390 (4)	C2B—C1B	1.400 (5)
O4B—C9B	1.426 (4)	C5B—C6B	1.373 (5)
O4A—C8A	1.383 (4)	C5B—H5B	0.9500
O4A—C9A	1.426 (4)	C6B—C1B	1.380 (5)
O1A—C7A	1.260 (4)	C6B—H6B	0.9500
O2A—C7A	1.279 (4)	C6A—C1A	1.383 (5)
O2A—H2A	0.83 (3)	C6A—H6A	0.9500
O1B—C7B	1.275 (4)	C9A—H9A1	0.9800
O2B—C7B	1.271 (4)	C9A—H9A2	0.9800
O2B—H2B	0.82 (3)	C9A—H9A3	0.9800
C4A—C5A	1.395 (5)	C8A—H8A1	0.9900
C4A—C3A	1.398 (4)	C8A—H8A2	0.9900
C4A—C7A	1.482 (5)	C8B—H8B1	0.9900
C7B—C4B	1.476 (5)	C8B—H8B2	0.9900
C4B—C5B	1.394 (5)	C9B—H9B1	0.9800
C4B—C3B	1.402 (4)	C9B—H9B2	0.9800
C2A—C3A	1.386 (5)	C9B—H9B3	0.9800
			
C2B—O3B—C8B	117.7 (2)	C5B—C6B—C1B	120.4 (3)
C2A—O3A—C8A	118.1 (2)	C5B—C6B—H6B	119.8
C8B—O4B—C9B	113.7 (3)	C1B—C6B—H6B	119.8
C8A—O4A—C9A	113.3 (3)	C6B—C1B—C2B	121.2 (3)
C7A—O2A—H2A	116 (3)	C6B—C1B—Br1B	118.9 (3)
C7B—O2B—H2B	124 (4)	C2B—C1B—Br1B	119.9 (2)
C5A—C4A—C3A	120.9 (3)	C1A—C6A—C5A	119.5 (3)
C5A—C4A—C7A	120.5 (3)	C1A—C6A—H6A	120.2
C3A—C4A—C7A	118.5 (3)	C5A—C6A—H6A	120.2
O2B—C7B—O1B	123.3 (3)	O4A—C9A—H9A1	109.5
O2B—C7B—C4B	117.5 (3)	O4A—C9A—H9A2	109.5
O1B—C7B—C4B	119.1 (3)	H9A1—C9A—H9A2	109.5
C5B—C4B—C3B	120.6 (3)	O4A—C9A—H9A3	109.5
C5B—C4B—C7B	119.9 (3)	H9A1—C9A—H9A3	109.5
C3B—C4B—C7B	119.4 (3)	H9A2—C9A—H9A3	109.5
O3A—C2A—C3A	124.7 (3)	O4A—C8A—O3A	113.1 (3)
O3A—C2A—C1A	116.6 (3)	O4A—C8A—H8A1	109.0
C3A—C2A—C1A	118.6 (3)	O3A—C8A—H8A1	109.0
O1A—C7A—O2A	123.3 (3)	O4A—C8A—H8A2	109.0
O1A—C7A—C4A	118.9 (3)	O3A—C8A—H8A2	109.0
O2A—C7A—C4A	117.8 (3)	H8A1—C8A—H8A2	107.8
C6A—C5A—C4A	119.2 (3)	O4B—C8B—O3B	113.0 (3)
C6A—C5A—H5A	120.4	O4B—C8B—H8B1	109.0
C4A—C5A—H5A	120.4	O3B—C8B—H8B1	109.0
C2A—C3A—C4A	119.8 (3)	O4B—C8B—H8B2	109.0
C2A—C3A—H3A	120.1	O3B—C8B—H8B2	109.0
C4A—C3A—H3A	120.1	H8B1—C8B—H8B2	107.8
C2B—C3B—C4B	119.7 (3)	C6A—C1A—C2A	122.0 (3)
C2B—C3B—H3B	120.1	C6A—C1A—Br1A	119.1 (3)
C4B—C3B—H3B	120.1	C2A—C1A—Br1A	119.0 (2)
O3B—C2B—C3B	124.8 (3)	O4B—C9B—H9B1	109.5
O3B—C2B—C1B	116.4 (3)	O4B—C9B—H9B2	109.5
C3B—C2B—C1B	118.8 (3)	H9B1—C9B—H9B2	109.5
C6B—C5B—C4B	119.3 (3)	O4B—C9B—H9B3	109.5
C6B—C5B—H5B	120.4	H9B1—C9B—H9B3	109.5
C4B—C5B—H5B	120.4	H9B2—C9B—H9B3	109.5
			
O2B—C7B—C4B—C5B	−8.5 (4)	C4B—C3B—C2B—C1B	0.0 (4)
O1B—C7B—C4B—C5B	170.4 (3)	C3B—C4B—C5B—C6B	1.7 (4)
O2B—C7B—C4B—C3B	172.9 (3)	C7B—C4B—C5B—C6B	−176.9 (3)
O1B—C7B—C4B—C3B	−8.3 (4)	C4B—C5B—C6B—C1B	−0.6 (4)
C8A—O3A—C2A—C3A	2.2 (4)	C5B—C6B—C1B—C2B	−0.8 (4)
C8A—O3A—C2A—C1A	−178.5 (3)	C5B—C6B—C1B—Br1B	176.5 (2)
C5A—C4A—C7A—O1A	−175.6 (3)	O3B—C2B—C1B—C6B	180.0 (3)
C3A—C4A—C7A—O1A	0.8 (4)	C3B—C2B—C1B—C6B	1.1 (4)
C5A—C4A—C7A—O2A	2.4 (4)	O3B—C2B—C1B—Br1B	2.7 (4)
C3A—C4A—C7A—O2A	178.9 (3)	C3B—C2B—C1B—Br1B	−176.2 (2)
C3A—C4A—C5A—C6A	−1.5 (4)	C4A—C5A—C6A—C1A	−0.4 (4)
C7A—C4A—C5A—C6A	174.8 (3)	C9A—O4A—C8A—O3A	−65.8 (3)
O3A—C2A—C3A—C4A	−180.0 (3)	C2A—O3A—C8A—O4A	−65.6 (3)
C1A—C2A—C3A—C4A	0.7 (4)	C9B—O4B—C8B—O3B	−74.1 (3)
C5A—C4A—C3A—C2A	1.3 (4)	C2B—O3B—C8B—O4B	−76.2 (3)
C7A—C4A—C3A—C2A	−175.1 (3)	C5A—C6A—C1A—C2A	2.5 (4)
C5B—C4B—C3B—C2B	−1.4 (4)	C5A—C6A—C1A—Br1A	−177.4 (2)
C7B—C4B—C3B—C2B	177.2 (3)	O3A—C2A—C1A—C6A	178.0 (3)
C8B—O3B—C2B—C3B	7.7 (4)	C3A—C2A—C1A—C6A	−2.6 (4)
C8B—O3B—C2B—C1B	−171.1 (3)	O3A—C2A—C1A—Br1A	−2.2 (4)
C4B—C3B—C2B—O3B	−178.8 (3)	C3A—C2A—C1A—Br1A	177.2 (2)

### Hydrogen-bond geometry (Å, º)

**Table:**

*D*—H···*A*	*D*—H	H···*A*	*D*···*A*	*D*—H···*A*
O2*A*—H2*A*···O1*B*	0.84 (5)	1.80 (5)	2.635 (4)	178 (5)
O2*B*—H2*B*···O1*A*	0.82 (5)	1.81 (5)	2.621 (4)	167 (5)
C8*B*—H8*B*2···O3*A*^i^	0.99	2.52	3.420 (4)	150

Symmetry code: (i) *x*−1, *y*+1, *z*−1.
